# Profiles of intercultural sensitivity of healthcare students: a person-centred approach

**DOI:** 10.5116/ijme.66dd.beb3

**Published:** 2024-09-26

**Authors:** Lilla Lucza, Tamás Martos, Viola Sallay, Tamás Simon, Anne Weiland, Peter Vermeir, Márta Csabai

**Affiliations:** 1 Doctoral School of Education, University of Szeged, Hungary; 2 Institute of Psychology, University of Szeged, Hungary; 3 Department for Internal Medicine & General Practice, Erasmus MC University Medical Center, The Netherlands; 4 Faculty of Medicine and Healthcare Sciences, Ghent University, Belgium; 5 Institute of Psychology, Department of Personality and Health Psychology, Károli Gáspár University of the Reformed Church, Hungary

**Keywords:** Intercultural sensitivity, empathy, healthcare students, latent profile analyses, medical education

## Abstract

**Objectives:**

We aimed to explore healthcare students' intercultural sensitivity profiles and their relationship with empathy to develop effective education methods that promote non-discriminatory patient care.

**Methods:**

We conducted a cross-sectional questionnaire study, involving a total of 508 international (n= 100) and local (n= 408) healthcare students in Hungary by convenience sampling. The survey included demographics, the Intercultural Sensitivity Scale, and the Interpersonal Reactivity Index. We applied latent profile analysis to identify distinct sensitivity profiles and used multinomial logistic regression to estimate the predictive power of several background variables on profile group membership.

**Results:**

A four-profile solution emerged: “Interculturally average” (n= 241), “Interculturally uncertain” (n= 76), “Interculturally sensitive” (n= 132), and “Interculturally refusing” (n= 54). The model (R^2^= 0.123; p= 0.001) revealed that psychology major tended to predict “uncertain” group membership (OR= 0.56, p= 0.08) and higher personal distress was a significant predictor of this group (OR=1.11, p= 0.002). Male gender (OR= 3.03, p= 0.001), medicine major (OR= 5.49, p= 0.01), lower perspective-taking (OR= 0.91, p= 0.007) and higher personal distress (OR= 1.09, p= 0.028) were identified as predictors of “refusing” group membership, compared to the “average” group.

**Conclusions:**

By exploring the ways students experience intercultural situations, a more personalized medical education can be developed with a special focus on vulnerable subgroups. For the “uncertain” group, the focus should be more on developing confidence, and intercultural experiences, whereas in the “refusing” group on strengthening empathy. In general, it can be useful to create mixed-gender, multidisciplinary, and intercultural learning environments.

## Introduction

As globalization and technological progress accelerate worldwide, cultural diversity creates a growing need for a higher level of understanding and sensitivity toward other cultures.^1^^-^^4^Cultural differences can challenge healthcare and lead to barriers for example, in the doctor-patient relationship.^4^^-^^7^ To address these challenges, in 2016, the WHO Regional Committee for Europe adopted the strategy and action plan for refugee and migrant health.^8^ To achieve healthcare systems that consider cultural diversity, without discrimination or stigma, healthcare students and employees must manage intercultural situations competently. Along with providing equal care to refugees and migrants, intercultural competence (IC) is also required to care for domestic minorities, such as ethnic, religious, or sexual, as well as to teach foreign students and support international research collaborations.^9^^-^^11^

Therefore, in recent years, an increasing number of publications have drawn attention to the importance of IC in medical education.^12^ There is currently no consistent definition of IC; it is a wide concept with many different perspectives in the literature, and its usage is highly discipline- and context-dependent.^10^^,^^12^ However, IC approaches focus not on the detailed acquisition of knowledge about other cultures but on the social, interpersonal, communicative, and action competencies developed through personal interaction.^12^^-^^14^ These personal interactions are particularly important in doctor–patient relationships, as IC is related to the anxiety and stress levels of professionals and their patients, their communication and satisfaction, and the quality of patient care.^15^^-^^22^ Members of these professions must be more competent in adopting culture-sensitive approaches than other disciplines.^16^^,^^23^

IC can be considered as a consistent set of learned attitudes, rules and behaviors that provides an ability to mobilize our inner resources (knowledge, emotions, skills) in order to successfully function within a cross-cultural situation.^3^^,^^12^ According to the model described by Chen and Starosta (1996), IC has three components. First, the cognitive component is defined as intercultural awareness, which refers to an enhanced understanding of the diverse characteristics of different cultures. Second, the affective component is described as intercultural sensitivity (ISE) and third, the behavioral component is defined as communicative competence itself. These are separate, yet closely related and mutually dependent concepts that interact.^1^^,^^2^

ISE (the affective component of IC) is an individual’s ability to develop positive emotions and be motivated toward understanding, appreciating, and accepting cultural differences. Authors have demonstrated that this component is crucial for enhancing and buffering intercultural awareness and a key determinant of higher IC values.^1^^,^^2^^,^^24^ Chen and Starosta identify six factors of ISE: self-esteem, self-monitoring, openness, involvement in interaction, nonjudgment, and empathy.^2^

Continuing with an important one of these six factors, an empathic practitioner can accurately perceive changes in a patient’s condition, successfully address problems during treatment, and achieve patient satisfaction and compliance.^25^^-^^27^ Empathy also has many advantages for physicians and future physicians, such as satisfaction with education, interpersonal and teamwork skills, few lawsuits and complaints, and low levels of stress and burnout.^28^^-^^32^

Empathy is a complex multidimensional construct, including individual cognitive and perspective-taking capabilities and emotional reactivity.^33^ Medical education might prioritize clinical facts, which negatively affect the development of medical students’ empathy in addition to a hidden curriculum that may dehumanize patients.^34^^-^^35^ Some studies concluded that empathy decreases as the number of years of education increases.^26^ However this has not been confirmed by a recent review.^36^ Alongside IS, the teaching and development of empathy skills are highly relevant in medical education, for which several different methods have been developed.^37^^-^^39^

More empathic people can more accurately assess others’ internal state, express their emotions, and understand intercultural situations.^33^ This is supported by the fact that one of Davis’ empathy questionnaire subscales (perspective-taking) showed a significantly positive correlation with ISE.^40^ Ekong and colleagues’ results also revealed that empathy and ISE are significantly connected. They suggest incorporating training strategies in communication skills curricula, including empathy and ISE.^38^ Menardo investigated ISE and mindfulness and found that the positive association between them was mediated by empathy level.^41^ Based on these, it is worth examining ISE and empathy together to understand how they are present in a population where both can contribute to the quality of patient care.

Our previous variable-oriented studies have already confirmed that, in addition to empathy, other variables may also play a prominent role in the context of ISE.^42^ Results showed that life experiences abroad and foreign language proficiency are significantly connected to ISE.^15^^,^^42^^-^^47^ We can  also note that females scored significantly higher than males, which is consistent with the results of several previous studies.^10^^,^^15^^,^^42^^,^^48^

Overall, more medical and educational research has been conducted in the broad area of IC, which we know is complex, challenging to measure, and influenced by many factors.^10^^,^^12^^,^^49^ However, examining the specific concept of ISE is rare in the literature on the development of medical or healthcare students.^15^^,^^23^^,^^50^^,^^51^ This is one research gap we addressed.  The other is that the international literature has highlighted the need for the development of IC and ISE in health education to provide nonjudgmental and nondiscriminatory health services, for which it is essential to understand the ISE characteristics and needs of this population.^52^^-^^55^ Some studies have already shed light on how to shape medical students’ undergraduate education to equip them with ISE or IC skills, but these are not fully complete and encourage further research and development.^56^^-^^58^

The present study was a part of the Medical Education on Medically Unexplained Symptoms and Intercultural Communication (MUSIC) Erasmus+ project which aimed to gather existing knowledge about IC and connecting interventions for new assessments and education programs to better prepare European healthcare providers for their daily encounters with culturally diverse, often ethnic minority patients.^42^^,^^59^^-^^61^  To effectively implement the project’s educational improvements, a necessary step was to conduct this needs and status assessment study of local and international healthcare students. With that, our aim is to explore and understand different ISE profiles of healthcare students and their relationships with empathy and some other variables (age, nationality, faculty, foreign language proficiency, and life experiences abroad) in more depth.

The person-oriented analysis we use is exploratory; therefore, formal preliminary hypotheses are not possible.^62^

However, we assume that we will identify one or more student group(s) that are more interculturally sensitive and less in need of development, as well as one(s) whose members are less sensitive and could benefit from targeted development. Presumably, information will also be obtained about the composition of groups of students with similar ISE profiles, so lessons will be learned on the most effective ways for development.

## Methods

### Study design and participants

The cross-sectional, quantitative study was conducted at a large state university in Hungary. More specifically, our data was collected from healthcare students studying at the University of Szeged, which has a rich international background and offers degree programs both in German and English to students from over 60 different countries around the world.

The convenience sample of voluntary participants consisted of 508 respondents (mean age: 22.5 years, SD = 3.13), 359 of whom identified themselves as female (70.7%) and remaining as male (29.3%). It is worthwhile to note that students in years 1–3, female and medical students completed the questionnaire at higher rates than students in years 4–6, male and psychology students. Approximately 502 respondents answered all the demographic questions (Table 1).

**Table 1:** Distribution of participants by field of study, gender, and year of study

Year of Study	Hungarian medical		International medical		Hungarian psychology		Other*		Sum
	female	male	female	male	female	male	female	male	
Yr 1	5	-	22	20	19	6	2	1	75
Yr 2	60	27	14	8	19	7	3	-	138
Yr 3	59	25	8	13	25	7	1	-	138
Yr 4	43	19	2	4	19	-	1	1	89
Yr 5	5	3	2	2	32	2	-	1	47
Yr 6	10	-	4	1	-	-	-	-	15
Sum	182	74	52	48	114	22	7	3	502

*Pharmacist / Nurse / Physiotherapist / Special Education

In addition to the 403 Hungarian medical and psychology students, 105 international (mostly German (n = 23, 21.9%), Iranian (n = 23, 21.9%), and South Korean (n = 9, 8.6%)) medical students, now studying in Hungary, were represented in the sample. Moreover, there were between one and seven respondents from 22 other nationalities, forming a very heterogeneous international sample.

In total, 124 students (24.4%) spoke one foreign language other than their mother tongue, 292 (57.5%) spoke two foreign languages and 92 (18.1%) had mastered three or more. Most participants, 63.8% (n = 324), had never spent more than three months living abroad, 22.6% (n = 115) had lived abroad once, and 13.5% (n = 69) had spent time in foreign countries twice or more than twice.

### Data collection

Our needs and status assessment survey started with an informed consent and a demographic section. Basic demographic data was collected, including age, gender, university, year of study, major, nationality, number of languages spoken besides the mother tongue, and how many times the respondent had lived abroad for more than three months. Then two other questionnaires were included, the intercultural sensitivity scale (ISS) and the interpersonal reactivity index (IRI) (which measures empathy), these are presented in more detail below.^33^^,^^40^

The ISS consists of 24 items (nine of which are negatively measured) and the authors identified five factors: interaction engagement (seven items, e.g., “I often give positive responses to my culturally different counterpart during our interaction”); respect for cultural differences (six items, e.g., “I respect the ways people from different cultures behave”); interaction confidence (five items, e.g., “I am pretty sure of myself in interacting with people from different cultures”); interaction enjoyment (three items, e.g., “I often get discouraged when I am with people from different cultures”); and interaction attentiveness (three items, e.g., “I try to obtain as much information as I can when interacting with people from different cultures”).^40^ Subjects are required to indicate on a 5-point Likert scale the extent to which they agree with the item statement. A score of 1 means “strongly disagree,” and a score of 5 means “strongly agree.”^40^

Finally, the IRI measures empathy on four subscales: the fantasy subscale, perspective-taking subscale, empathic concern subscale, and personal distress subscale.^33^ The fantasy subscale appears to tap the tendency to imaginatively transpose oneself into fictional situations (e.g., books, movies, daydreams). The perspective-taking subscale, which, on its face, reflects an ability or proclivity to shift perspectives—to step “outside the self”—when dealing with other people. The items comprising this scale refer to real-life instances of perspective-taking and not fictitious situations and characters. The empathic concern subscale assesses the degree to which the respondent experiences feelings of warmth, compassion, and concern for the observed individual. The personal distress subscale measures an individual’s feelings of fear, apprehension, and discomfort at witnessing the negative experiences of others. Each subscale has seven items, and the questionnaire consists of 28 items in total, nine of which measure negatively. Respondents are asked to indicate the extent to which each item describes them on a 5-point Likert scale.^33^

### Procedure

After a literature review and a search for appropriate and available measurement tools, the data collection was carried out between May and March 2019 and between February and April 2020, using online (Google Forms) and paper–pencil questionnaires. We made the questionnaire available in-person at students' various courses and in their library, but also online through their mailing lists and university learning platforms.

The research has been performed in accordance with the Declaration of Helsinki. On the first page of the questionnaire, participants were given the informed consent form, which they could accept by completing the paper questionnaire and submitting it to the data collection officer or by sending it in the case of the online questionnaire. The consent informed the students that their participation is completely voluntary, and they are free to decline without consequence. Moreover, that the study is anonymous, so we do not collect or retain any information about their identity, and the information provided by them will be used only for research purposes and will only be analyzed and published in aggregate form.  The ethical considerations of our study were approved by the Hungarian Joint Committee on Research Ethics in Psychology.

### Data analysis

Based on the data collected from the students, we performed a person-oriented analysis. The goal of person-oriented research is to uncover meaningful and distinct subgroups within a population. These subgroups may have different patterns, levels, or combinations of variables. By identifying these latent profiles, researchers can gain insights into different types of individuals or groups within a larger population.^62^

For all statistical tests of our study, a p-value smaller than 0.05 was established for determining significance. Before the person-oriented analysis, we tested the internal reliability of our measures and the internal correlations of the ISS subscales. We conducted these analyses using the statistical package for social sciences (SPSS), version 20.0 (IBM Corp., Chicago, IL).

The person-oriented analysis we used was Latent Profile Analysis (LPA) which  was conducted using Jamovi version 2.3.17 (The Jamovi project, 2022) with the module snowRMM (Seol, 2022) that utilizes the tidyLPA R-package.^63^ This model-based clustering technique was developed by Gibson.^64^^,^^65^ LPA identifies latent grouping variables by decomposing the covariance matrix to create subgroups that are assumed to be homogenous in their latent profiles. We interpret latent profiles based on their characteristic means on the indicator variables of ISS subscales. We tested the available latent profiles in all variance–covariance matrix types to choose the right fit based on multiple statistical fit indices. Better model fit was indicated by lower levels of the Bayesian information criterion (BIC), the Akaike information criterion (AIC), the sample size adjusted Bayesian information criterion (SSA-BIC), and the higher entropy value; as well as the significance result of the Lo-Mendel–Rubin adjusted likelihood ratio test (LMRT).^66^ We considered BIC and LMRT the most applicable indices for model fit.^67^

After running LPA, using SPSS version 20.0 and Jasp version 0.16. 3.0, cross tables were used to better understand the relationships between gender, major, nationality, and cluster membership. Furthermore, we tested the differences in age, foreign language proficiency, life experiences abroad, and empathy levels between the ISS clusters with one-way ANOVAs. The statistical predicting effect of these abovementioned series of independent variables (controlled for each other) was also tested in a multinomial logistic regression model, which deals with one nominal response variable (ISS clusters) having more than two categories.

## Results

First, we examined the internal reliability of the scales used. The main scales of the questionnaires, IRI and ISS, had good Cronbach’s alpha values of above 0.8. In most cases, the subscales’ internal consistency estimates were considered acceptable to excellent (ranging from 0.71 to 0.88), except for the moderate estimates of the IRI empathic concern (0.54) and ISS attentiveness (0.60) subscales.

Second, before the LPA, Pearson correlations were performed to determine the degree of connection between the ISS subscales. If the correlation is too strong (r > 0.7) between two subscales, it may raise the question of whether it is worth including the subscales separately as indicator variables for the later LPA test. The ISS subscales were all significantly and positively correlated, only the interaction confidence and interaction enjoyment subscales showed an elevated value compared to the ideal for estimating latent profiles (r(503) = 0.73, p = 0.001) (Table 2). Nevertheless, given the good internal reliability of the two subscales, it was worth including them both separately in the LPA (Table 2).

We used the five ISS subscale scores (interaction engagement, respect for cultural differences, interaction confidence, interaction enjoyment, and interaction attentiveness) as indicator variables for the LPA. For best-fit indices, theoretical expectations, and substantive interpretability, finally, we used profile-invariant unrestricted variance–covariance matrix specification (eq, eq). Solutions with two to six latent profiles and the corresponding fit indices were computed. The inspection of the fit indices suggested that best-fitting solutions were the 4-class solution or the 6-class solution. See the detailed indices in Appendix A. Considering the lowest BIC value, interpretability, and the appropriate latent profile group sizes for further calculations, the 4-cluster solution was chosen (AIC = 6125; BIC = 6286; SSA-BIC = 6165; Entropy = 0.64; LMRT = 51.40, p = 0.001).

Table 3 presents the subgroup profiles using their means from the initial ISS subscale Z-scores. We used these standardized Z-scores to interpret the four distinct clusters. The first latent profile group has the largest number of participants (n = 241) and is easy to interpret because all ISS subscales are close to the medium Z-score. The group of students who experience intercultural situations this way are called “Interculturally Average (IA).”

**Table 2:** Internal Correlations of ISS Subscales

Variables		ISS Respect	ISS Confidence	ISS Enjoyment	ISS Attentiveness
ISS Engagement	Pearson Correlation (r)	.57	.45	.42	.52
	p	.001	.001	.001	.001
	N	504	504	504	505
ISS Respect	Pearson Correlation (r)		.16	.33	.44
	p		.001	.001	.001
	N			504	504
ISS Confidence	Pearson Correlation (r)			.73^*^	.27
	p			.001	.001
	N			503	504
ISS Enjoyment	Pearson Correlation (r)				.237
	p				.001
	N				504

p = probability value (significance level); N = number of participants in the actual correlation; *stronger correlation coefficient (r) than ideal for estimating latent profiles

In the second latent profile group, the “Interculturally Uncertain (IU)” group of students (n = 76) scored around the medium range for interaction engagement, attentiveness, and respect for cultural differences; however, this group had low scores for confidence and enjoyment of interaction. The third group, with the second highest number of participants (n = 132), stands out as they scored higher than the medium range on all the ISS subscales. This group was named the most successful “Interculturally Sensitive (IS)” group. In contrast, the last latent profile group is a smaller but more distinct subgroup (n = 54) where all the ISS subscales score low, with only the interaction confidence approaching the lowest value in the medium range. This less sensitive group of students was referred to as “Interculturally Refusing (IR).”

Latent profile membership was cross-tabulated by gender, nationality, and university major. For gender, the overall Chi-square test revealed a significant difference in the distribution of membership ratios (χ^2^(3) = 24.64, p = 0.001). Female respondents were more likely to be members of IU and IS, whereas males had a higher probability of being members of IR. Regarding majors, the test also indicated a significant difference in membership ratio distribution (χ^2^(3) = 23.85, p = 0.001). Psychology students were more likely to be members of IU, whereas medical students had a higher probability of being members of IR. However, in the case of nationality, the overall Chi-square test did not reveal a significant difference in the distribution of membership ratios (χ^2^(3) = 6.00, p = 0.11).

One-way Analysis of Variance (ANOVA) tests were applied to explore the possible differences between the latent profile groups regarding the participants’ age (F_(3.5)_ = 2.09, p = 0.10), foreign language proficiency (F_(3.5)_ = 5.28, p = 0.001), life experiences abroad (F_(3.5)_ = 2.84, p = 0.04) and empathy main scale scores (F_(3.5)_ = 7.32, p = 0.001). The same way, latent profile groups were also compared across the subscales of the empathy main scale, namely the fantasy subscale (F_(3.5)_ = 2.16, p = 0.09), the perspective-taking subscale (F_(3.5)_ = 13.59, p = 0.001), the empathic concern subscale (F_(3.5)_ = 3.22, p = 0.02) and the personal distress subscale (F_(3.5)_ = 17.98, p = 0.001) were examined. As can be read from the results, we found significant differences between the four student groups in all the abovementioned variables, except age and the fantasy subscale of IRI.

Post-hoc tests with Bonferroni adjustment revealed the details of the underlying causes of the group differences. The average number of foreign languages spoken is significantly greater in the IS (M = 2.16, SD = 0.75) than in the IA (M = 1.92, SD = 0.71) and IU (M = 1.79, SD = 0.72) groups. The average amount of life experience abroad is also significantly higher in IS (M = 0.68, SD = 0.90) than in IU (M = 0.33, SD = 0.62). The mean of the IRI perspective-taking subscale is significantly higher in IS (M = 20.36, SD = 4.39) than in IA (M = 18.66, SD = 4.63) and lower in IR (M = 15.91, SD = 3.91) than in the other three groups. The mean of the IRI empathic concern subscale is significantly higher in the IU and IS groups (M = 18.25, SD = 3.57 and M = 17.97, SD = 3.73) than in the IR (M = 16.33, SD = 3.63) group. The last subscale of IRI, personal distress is significantly lower in IS (M = 10.23, SD = 5.33) than in IA (M = 12.41, SD = 4.34) and IR (M = 12.57, SD = 3.76), but significantly higher in IU (M = 15.23, SD = 5.04) than in the other three groups. Finally, looking at the overall empathy means, IU (M = 72.55, SD = 11.59) scored significantly higher than IA (M = 67.58, SD = 12.01) and IS (M = 67.60, SD = 12.93), whereas IR (M = 62.50, SD = 11.25) scored significantly lower than all other three groups.

We ran a multinomial logistic regression analysis to understand the predictive power of the variables used in cross tabulations and ANOVAs on latent profile membership. We chose IA group as the reference category since this was the most numerous subgroup; thus, it represented a common type. Our model was significant (χ^2^(30) = 145, R^2 ^= 0.123, p = 0.001). Age, nationality, life experiences abroad, fantasy and concern subscales of empathy, and the overall empathy score did not predict any profile memberships, which partially confirms our earlier results. Significant unique contributions were made by all other variables, the followings found to have significant predictive power for being a member of at least one of the three groups, compared to the reference group.

**Table 3:** Comparison of the latent profile groups along the ISS subscale average scores

Variable	IA “Interculturally Average”		IU “Interculturally Uncertain”		IS “Interculturally Sensitive”		IR “Interculturally Refusing”		F* overall	df	p
	m (SD)	95% CI lower, upper	m (SD)	95% CI lower, upper	m (SD)	95% CI lower, upper	m (SD)	95% CI lower, upper			
ISS Engagement	.05 (.77)	-.05, .15	-.27 (1.22)	-.55, .01	.57 (.69)	.45, .69	-1.22 (1.03)	-1.50, -.94	57.97	3,499	.001
ISS Respect	.12 (.65)	.04, .20	.01 (.68)	-.14, .17	.56 (.52)	.56, .74	-2.12 (.69)	-2.31, -1.94	254.14	3,499	.001
ISS Confidence	-.08 (.71)	-.17, .01	-1.26 (.71)	-1.42, -1.10	1.02 (.52)	.93, 1.11	-.39 (.89)	-.63, -.15	188.87	3,499	.001
ISS Attentiveness	-.10 (.90)	-.21, .01	-.38 (.85)	-.57, -.18	.78 (.69)	.66, .89	-.88 (1.03)	-1.16, -.60	61.15	3,499	.001
ISS Enjoyment	.17 (.457)	.09, .24	-1.50 (.59)	-1.64, -1.37	.91 (.40)	.84, .98	-.86 (.96)	-1.12, -.59	311.76	3,499	.001
n	241		76		132		54				

ISS Engage. = interaction engagement, ISS Resp. = respect for cultural differences, ISS Conf. = interaction confidence, ISS Atten. = interaction attentiveness, ISS Enjoy. = interaction enjoyment

*all pairwise comparisons are significantly different (p < .05) using Bonferroni procedure in post-hoc test

Psychology major tended to predict IU group membership (OR = 0.56, CI = 0.29 – 1.01, p = 0.08); that is, psychology students compared to medical students were somewhat more likely to be members of IU than IA. A higher level of empathy’s personal distress factor was also a significant predictor of IU membership (OR = 1.11, CI = 1.04 – 1.18, p = 0.002). A higher level of foreign language proficiency was predictive of being a member of the IS group (OR = 1.51, CI = 1.09 – 2.01, p = 0.014), as was the higher level of perspective-taking (OR = 1.08, CI = 1.03 – 1.14, p = 0.004). A lower level of personal distress was also indicative of membership in IS (vs. IA) (OR = .89, CI = 0.84 – 0.93, p = 0.001). Finally, the IR group membership was predicted by gender (OR = 3.03, CI = 1.55 – 5.92, p = 0.001) and major (OR = 5.49, CI = 1.51 – 20.02, p = 0.01); male respondents and medical students (when controlling for all other characteristics) were more likely to be members of IR (vs. IA). At the same time, a lower level of perspective-taking (OR = 0.91, CI = 0.84 – 0.97, p = 0.007) and a higher level of personal distress (OR = 1.09, CI = 1.01 – 1.17, p = 0.028) also significantly predicted membership.

## Discussion

Our study aimed to explore and understand intercultural sensitivity profiles of healthcare students and their relationships with empathy in as much depth as possible. Additionally, we sought to understand the composition of groups of students with similar ISE profiles to determine the most effective ways to develop their intercultural competences.

Using an innovative person-oriented analysis, the results captured four latent ISE profiles among healthcare students using ISS subscales as indicator variables (interaction engagement, respect for cultural differences, interaction confidence, enjoyment, and attentiveness). The first latent profile group has the most students (241) whose ISS subscale scores are all very close to the medium Z-score, called the ‘Interculturally Average (IA)’ group. Our univariate statistical tests showed that the average number of foreign languages spoken and the mean of the empathy subscale perspective-taking are significantly lower in this group than in the particularly sensitive group, at the same time their scores are higher on the personal distress subscale of empathy. Thus, we consider that they stand relatively confidently between the highly sensitive and refusing behavioral modes in intercultural situations. If they are motivated, they could further develop their ISE by learning foreign languages, improving their perspective-taking skills, and reducing their distress. We used this larger group as a reference in our multivariate statistical test when we examined the remaining three groups.

The second latent profile group is the ‘Interculturally Uncertain (IU)’. Seventy-six students scored around the medium range for interaction engagement, attentiveness, and respect for cultural differences. However, they scored lower for confidence and enjoyment during intercultural interactions. Univariate analysis indicated that female and psychology major respondents were more likely to be members of this group than male and medical students. Foreign language proficiency and life experiences abroad were lower in the IU group than in the interculturally sensitive group. The scores for overall empathy, perspective-taking, and empathic concern were highest in the IU group than in others, but only personal distress was significantly greater in this group. Our multinominal regression confirmed that IU group membership was predicted by major and the personal distress factor of empathy.

Regarding these results, this is one group of students for whom targeted ISE development may be more recommended. In their case, empathy is so high that it may even cause personal distress. Development should focus on strengthening their self-confidence in intercultural situations in a personal and experience-based way. Intercultural study groups, community programs, study abroad, or language courses (even online), could all be useful for this group.^15^^,^^43^^-^^47^ The aim is for them to develop through positive reinforcement and gradually learn to enjoy these situations. It is also worth reflecting on why female and psychology students were overrepresented in this group. One possibility is that different social and gender norms and expectations of female and their insecurities may be more legitimate than male norms in the medical profession, which is considered highly prestigious and male-dominated, with a hidden curriculum.^35^^,^^68^^-^^71^ Future research may consider examining self-esteem and anxiety as possible predictors of membership in this group.^1^^,^^40^^,^^45^

A previous study by our research team examined the teachers of healthcare students.^60^ A common point among the teachers was that intercultural situations support students’ ability to broaden their worldview, thus enabling them to act without judgment. However, creating common principles of intercultural communication and expressing empathy was found to be challenging, even among the teachers. Hence, the “teach the teachers” approach may be worth considering. Moreover, they stressed that psychologists have a crucial role to play in gaining a deeper understanding of patients’ experiences. For this to happen, however, it is not enough to perceive a large proportion of psychologists as IU group members, strengthening their intercultural confidence is necessary.

With 132 students, the ‘Interculturally Sensitive (IS)’ group was the second largest. This group stood out as they scored higher than the medium range on all ISS subscales. Female respondents were more likely to fall into this group than males, despite the multinominal logistic regression not confirming gender as a predictive variable. The average amount of time spent abroad, and the number of foreign languages spoken were significantly greater in this group than in the uncertain group. For the empathy scale and some of its subscales, higher scores were obtained in this group than in other groups. However, the personal distress subscale of empathy scored lower for the IS group than for the three other groups. According to the multinomial logistic regression, foreign language proficiency, perspective-taking, and overall empathy were positively predictive of being an IS group member, whereas personal distress was negatively predictive.

The results indicate that this group might require the least amount of development. Its members have intercultural experiences and language skills and are not affected by debilitating stress or anxiety in intercultural situations. Additionally, their empathy is high, and they have the ability to change their perspective when dealing with others. Their behavior serves as a positive example for their fellow students during intercultural interaction developmental tasks.

Finally, the last student group is a relatively smaller separate subgroup with 54 participants. They have the lowest scores of all ISS subscales. Only the interaction confidence subscale approaches the lowest value in the medium range. This less sensitive group of students has been referred to as ‘Interculturally Refusing (IR)’. Male and medical students were more likely to belong to this group than female and psychology students. The distinguishing features of this group are that the means of the empathy scale, the perspective-taking, and the empathic concern subscales are significantly lower than the other groups. However, they scored higher than the sensitive group on the personal distress subscale of empathy. Our regression model also confirmed that being a member of this group was predicted by gender and major as well as by a low level of perspective-taking and greater personal distress. Open-mindedness could be another factor that may predict membership in the IR group and would be interesting to examine in the future.^1^^,^^2^

Males’ and medical students’ lower empathy values may also have contributed to the shift in gender and major ratios in this case. Social expectations may have influenced this result. In addition, there may be differences between the training in medical and psychology majors in terms of practicing and showing empathy, and a hidden curriculum may also reinforce the expected confidence for medical students.^35^^,^^70^ Members of this group might change perspective less easily, and their interactions are not as free of negative feelings and distress as those of the sensitive group, despite rating themselves as of average confidence in intercultural situations. In any case, it is certain that development may be more justified for this group of students, but in a slightly different way than for the uncertain group.

Empathy development, notably perspective-taking, could be an outstanding goal for the IR group. Empathy development training and courses aimed at health students are fundamentally needed, and with proper methods, succeed.^37^^,^^39^ It may be worthwhile to include practices that help them accept their negative feelings and insecurities regarding these interactions. There might be a place for both an intercultural experience-based approach (similar to the uncertain group), but training for awareness on this ground can also be useful by developing knowledge of the customs of other cultures, bringing them closer to the students, and discussing them. It can be fruitful to carry out situational exercises where they put themselves in the shoes of a person from another culture and practice empathetic communication.^37^^,^^39^

However, our research has several limitations that must be considered while interpreting the results. First, cross-sectional data collection does not allow us to infer causal relationships and can only provide a snapshot, whereas ISE is a dynamic process. Furthermore, the groups’ distribution may not be representative due to sampling. The online survey data collection also included the shortcomings of self-report methods, and we relied only on Likert-scale ratings in our analysis. Finally, in two instances, ISS subscales’ α coefficients were lower than optimal, and low internal consistency may have affected the results.

## Conclusions

To summarize, we identified two student groups (IU and IR) that seemed to be in greater need of ISE improvement. In the IU group, the focus should be more on developing confidence, foreign language skills, and intercultural experiences, whereas in the IR group, the focus should be on strengthening empathy, perspective-taking, attentiveness, engagement, and respect. It would be valuable if students could be screened by self-questionnaire and offered training programs considering their ISE types and distinct needs. This would allow us to move toward more personalized educational and developmental opportunities. A training curriculum development subprogram is being implemented, that tries to follow this approach.

Furthermore, we can claim based on our regression model, that gender, major, foreign language proficiency, and empathy factors are significant predictors of ISE types. It may be useful to create mixed gender, major, and nationality study groups, and to promote foreign language learning, interdisciplinary and intercultural cooperation, so that students have mutually enhanced experiences. Moreover, supporting and promoting study trips abroad and learning foreign languages may also be developmentally beneficial.^11^^,^^47^

It could be beneficial for future research to compare the healthcare student population with other student populations to have a more comprehensive understanding of intercultural sensitivity and intercultural competence. This might help in identifying the most appropriate developmental approaches. However, starting with healthcare students is reasonable since members of healthcare professions must be more competent in adopting culture-sensitive approaches than other disciplines.^16^^,^^23^ In conclusion, our results provide valuable insights into the types of intercultural sensitivity of students preparing for healthcare professions, specific areas of development, and best options for an effective developmental process.

### Acknowledgements

The authors would like to thank Katalin Barabás, MD, PhD (University of Szeged) for her valuable support throughout the study.

###  Conflict of Interest

The author declares that there is no conflict of interest.

## Supplementary material

Supplementary file 1 Appendix A. Fit indices of the latent profile analyses with different profile numbers
