# Supplementary material for: Profiles of intercultural sensitivity of healthcare students: a person-centred approach
Source: Int J Med Educ. 2024 Sep 26;15:113–23. doi: 10.5116/ijme.66dd.beb3 (PMC11687379; doi:10.5116/ijme.66dd.beb3)
Supplement: Supplementary file 1 — Appendix A. Fit indices of the latent profile analyses with different profile numbers [file ijme-15-113-S1.pdf]

Appendix A

Fit indices of the latent profile analyses with different profile numbers

No. of profiles	AIC	BIC	SSA-BIC	Entropy	LMRT	p
2	6180	6290	6207	.77	66.26	.001
3	6165	6300	6198	.77	27.43	.001
4	6125	6286	6165	.64	51.40	.001
5	6123	6309	6169	.63	14.06	.158
6	6076	6287	6129	.77	58.82	.001

AIC = Akaike information criterion, BIC = Bayesian information criterion, SSA-BIC = sample size adjusted Bayesian information criterion, LMRT = Lo-Mendel–Rubin adjusted likelihood ratio test
